# Real-Time Fluorescent Cholangiography by Intrabiliary Indocyanine Green Administration With Near-Infrared Laparoscopy in Major Hepatectomy

**DOI:** 10.7759/cureus.40769

**Published:** 2023-06-21

**Authors:** Naoya Sasaki, Yusuke Okamura, Ryuta Nishitai

**Affiliations:** 1 Surgery, Shizuoka City Shizuoka Hospital, Shizuoka, JPN; 2 Surgery, Kyoto Katsura Hospital, Kyoto, JPN; 3 Surgery, Hyogo Prefectural Amagasaki General Medical Center, Amagasaki, JPN

**Keywords:** intrahepatic bile duct imaging, laparoscopic hepatobiliary surgery, intraoperative fluorescent cholangiography, near-infrared laparoscopy, indocyanine green fluorescence imaging

## Abstract

Background and purpose: Biliary injury is a severe complication that can be associated with liver surgery. Intrahepatic biliary anatomy can be evaluated using magnetic resonance cholangiopancreatography and X-ray cholangiography; however, an intraoperative real-time bile duct visualization method has not yet been reported. This study aimed to demonstrate the availability of real-time fluorescent cholangiography (FC) by intrabiliary indocyanine green administration with near-infrared laparoscopy in major hepatectomy.

Methods: The optimal concentration of indocyanine green (ICG) solution was examined ex vivo. The fluorescence intensity of the ICG solution and its mixture with bile was measured. Using a clinical trial model, ICG solution was injected into the cystic duct, followed by near-infrared laparoscopy performed during hepatectomy.

Results: The optimal concentration of ICG solution for FC was between 0.01 and 0.05 mg/mL. Three different laparoscopic systems were used in three hepatectomy cases. In all cases, the fluorescence of the intrahepatic bile ducts in the Glissonian sheath was clearly visualized using the near-infrared laparoscopic system. A small piece of tissue prevented the bile glow; thus, exposure of the Glissonian sheath was necessary for clear FC. This procedure also detected bile leakage from the cut surface of the liver.

Conclusions: Intrabiliary ICG administration and near-infrared laparoscopy enabled real-time intrahepatic FC during major hepatectomy.

## Introduction

Biliary injury is a severe complication that can be associated with liver surgery. Unrecognized bile leaks may cause life-threatening complications, such as infection, biliary stenosis, and liver failure. In a previous large cohort study, the incidence of bile leak after hepatic resection ranged from 1.7% to 12%. Biliary injury is most common when resection is performed for lesions near the hilus; this failure is most likely after right-sided hepatectomy, in which the anatomy of the right bile ducts is variable in the hilar region [1]. There are several modalities to evaluate intrahepatic biliary anatomy, such as contrast-enhanced computed tomography (CT) and magnetic resonance cholangiopancreatography (MRCP), and three-dimensional images can be constructed from these data to predict the bile duct and blood vessels that appear in the resection plane of the liver during hepatectomy. However, these imaging techniques are only preoperative simulations, which often do not coincide with the intraoperative appearance of the ever-changing surgical field.

Intraoperative cholangiography using a contrast agent and X-ray can show the most current information on the intrahepatic biliary tree. It is effective to confirm the cut line of the bile duct during the operation. However, the operation must be interrupted for a certain period so that radiographs may be taken and the image confirmed. In addition, frequent cholangiography is not preferable from the perspective of radiation exposure. Furthermore, in laparoscopic hepatectomy, it is difficult to obtain a clear radiograph according to the surgical position, such as the left lateral decubitus position, for the right hepatectomy. A real-time bile duct visualization technique should be used to avoid an unexpected biliary injury. If it is less invasive and inexpensive, it is even better.

Indocyanine green (ICG) has long been widely used for the evaluation of liver function. When injected intravenously, it rapidly binds to plasma proteins and is carried by the bloodstream, entirely extracted by the liver, and excreted into the bile. It is useful to estimate remnant liver function following major hepatectomy, especially those performed for living donor liver transplantation or hepatectomy for cirrhotic liver, as these patients are at risk of postoperative liver failure. ICG also has the property of being a fluorescent material and can be observed using a near-infrared (NIR) camera. Based on this property, it is used in the field of hepatobiliary surgery to visualize extrahepatic bile ducts, hepatic intersegmental borders, and liver tumors. There have been many reports on extrahepatic fluorescent cholangiography (FC) in cholecystectomy. However, few reports on intrahepatic FC in hepatectomy exist. One of the reasons for this, assumably, is the low contrast of fluorescence between the bile duct and liver parenchyma because, when administered intravenously, ICG first accumulates in hepatocytes and remains there for a long time. This study aimed to demonstrate an intraoperative real-time intrahepatic FC during major hepatectomy using a NIR endoscopic system. We hypothesized that direct intrabiliary administration of ICG is effective in visualizing the intrahepatic bile duct with high contrast.

As a preliminary experiment, we examined the optimal concentration and condition of the ICG solution ex vivo. Subsequently, we also performed intraoperative FC during hepatectomy in a clinical trial.

## Materials and methods

Determination of the concentration of the ICG solution

ICG (DIAGNOGREEN®, Daiichi Sankyo, Tokyo, Japan) was diluted with normal saline to several concentrations, and the fluorescence of the samples was recorded with an infrared camera (pde-neo®, photodynamic eye-neo, Hamamatsu Photonics K.K., Hamamatsu, Japan). A standard (ICG 0.01 mg/mL) and each target sample were placed side-by-side in the center of the visual field to compare their fluorescence, as the sensitivity of this camera is uneven between its center and periphery. Pictures of each pair were examined with the image analysis software Image J (National Institutes of Health, Bethesda, Maryland, USA) to measure the strength of the fluorescence. The fluorescence of the 0.01 mg/mL ICG sample was set as a standard. The fluorescence intensity of each sample was quantified relative to the standard value, and the experiment was repeated thrice.

It is known that the fluorescence of ICG is enhanced when it binds to serum or bile proteins [2]. In this study, the ICG solution and bile were mixed in equal amounts, and the fluorescent intensity of each sample was measured. The bile was taken from gallbladders with no infection provided by the patients who underwent laparoscopic cholecystectomy for gallstones or adenomyomatosis with written consent for experimental use. This experiment was repeated five times. The difference in fluorescent strength was statistically analyzed using a paired t-test.

Clinical trial

This experiment was designated a clinical trial because the intrabiliary administration of ICG is considered off-label drug use. The clinical trial was approved by the Kyoto Katsura Hospital Ethics and Clinical Research Review Committee, Kyoto, Japan.

The maximum concentration of the ICG solution was 0.05 mg/mL. The ICG solution was injected into the cystic duct or drainage tube placed in the biliary tract, up to 20 mL. Intravenous administration has been widely used, and its dosage is usually 0.5 mg/kg body weight with a concentration of 2.5-5.0 mg/mL. Therefore, the intrabiliary administration method was assumed to have no safety problems. Patients with hypersensitivity to ICG or iodine were excluded from this trial because ICG contains a small amount of iodine. This study targeted patients who underwent hepatectomy, fenestration of liver cysts, and cholecystectomy. Only patients who had been given a detailed explanation regarding this study and provided written consent had FC during their surgery.

Intraoperative fluorescent cholangiography

Based on the result obtained by the ex vivo experiment, ICG solution (0.01-0.05 mg/mL) was injected intrabiliarily through a tube placed into the cystic duct. To make the ICG glow effective, a small amount of bile was aspirated into a syringe, mixed with the ICG solution, and subsequently injected into the biliary tract. The injection was stopped when high pressure was felt through the plunger. An ICG injection was performed after exposure to the hilar Glissonian sheath. The ICG glow in the biliary tract in the hepatoduodenal ligament and hilar Glissonian sheath was observed using a NIR laparoscopy system. The NIR system was VISERA ELITE II (Olympus Medical Systems, Hamburg, Germany) for case one, laparoscopic extended medial segmentectomy, and IMAGE1 S™ 4U RUBINA (KARL STORZ, Tuttlingen, Germany) for case two, laparoscopic anterior segmentectomy. Case three was performed with a planned, open approach. After a large hemangioma in segment four was enucleated, FC and leak tests were performed using the 1688AIM 4K platform (Stryker Corporation, Kalamazoo, Michigan, USA).

## Results

Determination of the concentration of the ICG solution

**Figure 1:**
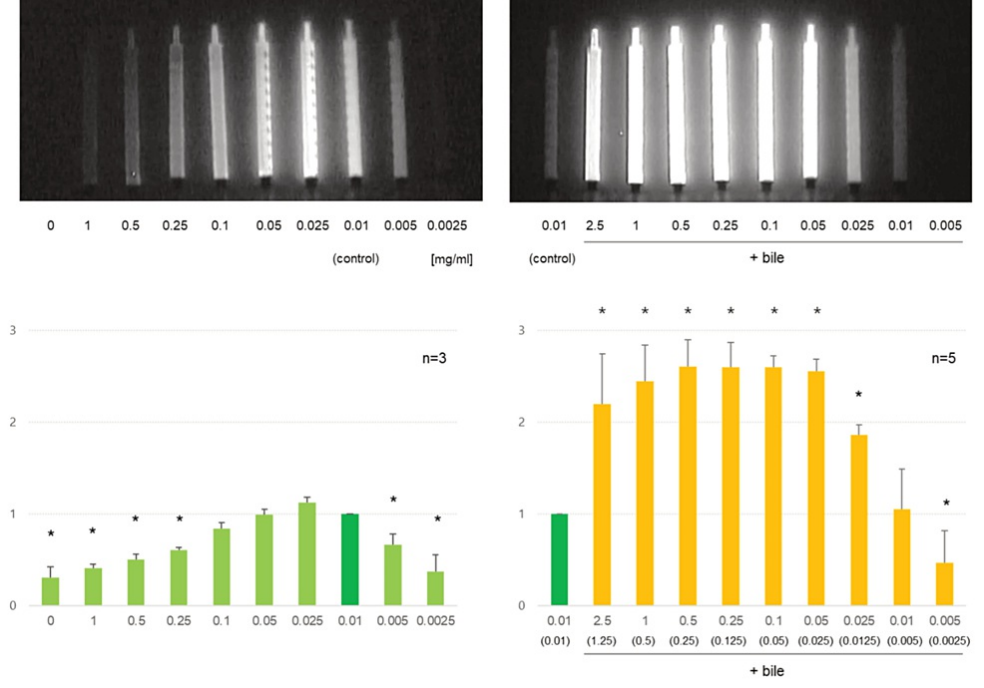
The fluorescence intensity of the ICG solution Left column: ICG dilution and fluorescent intensity. ICG is diluted with normal saline to various concentrations and recorded with an infrared camera. The fluorescent intensities are quantified as relative values based on the fluorescence of the control (0.01 mg/mL ICG solution). * Significantly different from the control (t-test, p< 0.05). Right column: ICG and bile mixture and fluorescent intensity ICG solution and bile are mixed equally, and the fluorescent intensity is quantified. The number in the bracket refers to the final concentration of ICG. ICG: indocyanine green

The fluorescence intensity of ICG solutions at various concentrations was measured using an infrared camera. ICG intensity was maximum in a 0.025 mg/mL solution; it was 1.13 times stronger than that of the standard (0.01 mg/mL). A strong glow was observed at a concentration of 0.01-0.1 mg/mL (p>0.05). Fluorescent staining was weakened at both high (≥ 0.25 mg/mL) and low concentrations (≤ 0.005 mg/mL) (Figure 1, left column; Video 1).

**Video 1:** The fluorescence intensity of the ICG solution Indocyanine green (ICG) was diluted with normal saline to various concentrations and recorded with an infrared camera. Second, ICG solutions and bile were mixed equally, and the fluorescence was recorded. A standard (ICG 0.01 mg/mL) and each target sample were placed side-by-side to quantify their fluorescent intensities.

Second, the fluorescence intensity of the ICG-bile mixture was measured. When mixed with an equal volume of bile, ICG fluorescence was enhanced at 0.025-2.5 mg/mL (the final concentration was 0.0125-1.25 mg/mL). It was more than doubled at 0.05-2.5 mg/mL (Figure 1, right column, Video 1).

Based on these results, the optimal ICG concentration for FC was determined to be approximately 0.025 mg/mL. Because the enhancement began to plateau at 0.05 mg/mL, the maximum ICG concentration for the clinical trial was set to be 0.05 mg/mL.

To investigate the relationship between the mixture fraction and fluorescent intensity, 0.025 mg/mL ICG solution and bile were mixed in varying proportions. Strong glows were observed in a wide range of ICG-bile proportions between 10%-90% (Appendix 1). It was confirmed that a strict preparation in mixed proportions was not necessary at the time of intraoperative FC. We chose 0.01 mg/mL, 0.025 mg/mL, and 0.05 mg/mL ICG concentrations for the clinical trial.

Intraoperative fluorescent cholangiography

Case One

**Figure 2:**
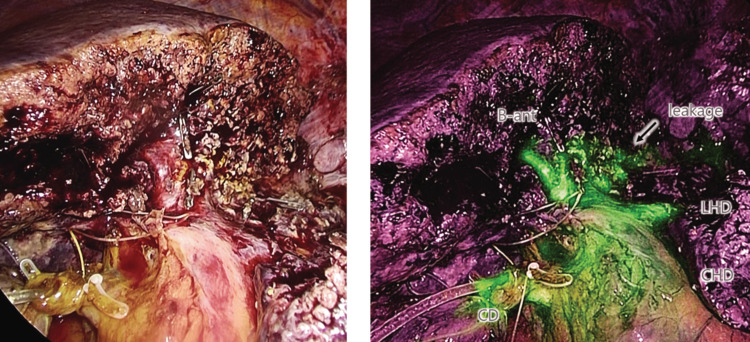
Intraoperative cholangiography during laparoscopic hepatectomy (Segments 4+8) After resection of the specimen, ICG solution (0.01 mg/mL) is injected into the cystic duct. Fluorescence in the bile ducts is then observed with NIR laparoscopy (VISERA ELITE II (Olympus Medical Systems, Hamburg, Germany). The common hepatic duct left, the right hepatic ducts, and the intrahepatic bile ducts of the anterior segment were clearly visualized. Bile leakage from the cut surface was observed. (Left) white light mode; (Right) infrared mode CD: cystic duct; CHD: common hepatic duct; LHD: left hepatic duct; B-ant: bile ducts of the anterior segment; ICG: indocyanine green; NIR: near-infrared

A 74-year-old man with non-alcoholic steatohepatitis had hepatocellular carcinoma (15 mm, single) in segments four and eight (S4/8), for which a laparoscopic extended medial segmentectomy was performed. After the specimen was resected, ICG solution (0.01 mg/mL) was injected into the biliary tract via the cystic duct. ICG showed fluorescence immediately after injection and displayed a stronger glow when mixed with bile. The right and left hepatic ducts and bile ducts of the anterior segment were visualized, and bile leakage from the cut surface of the liver was observed (Figure 2).

The leaked bile glow was removed using saline. After the closure of the bile leakage with a surgical clip, a leak test was performed, and no leakage was detected.

Case Two

**Figure 3:**
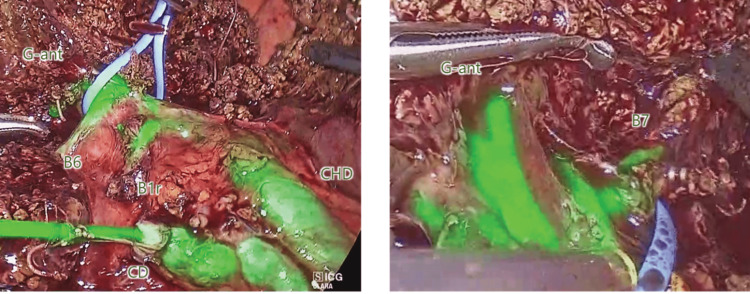
Intraoperative cholangiography during laparoscopic anterior segmentectomy After the major portal fissure is split, the Glissonian pedicle of the anterior segment is isolated with a vessel tape. ICG solution (0.05 mg/mL) is injected into the cystic duct. Fluorescence in the bile ducts is observed with NIR laparoscopy (IMAGE1 S™ 4U RUBINA (KARL STORZ, Tuttlingen, Germany). The extrahepatic and intrahepatic bile ducts of the anterior segment, posterior segment (B6, B7), and caudate lobe (B1r) are clearly visualized. CD: cystic duct; CHD: common hepatic duct; G-ant: Glissonian pedicle of the anterior segment; ICG: indocyanine green

The patient was a 77-year-old man with a metastatic liver tumor from colon cancer in segment eight (S8. 26 mm, single). A laparoscopic anterior segmentectomy was performed. The major portal fissure was split and exposed to the Glissonian sheath of the anterior segment. ICG solution (0.05 mg/mL) was injected into the biliary tract, the left and right hepatic ducts and their branches, the bile ducts of the anterior segment, segment six (B6), and segment seven (B7), respectively (Figure 3).

While watching the glow of the bile in the Glissonian sheath, the anterior branch was transected safely without any damage to the posterior branches.

Case Three

**Figure 4:**
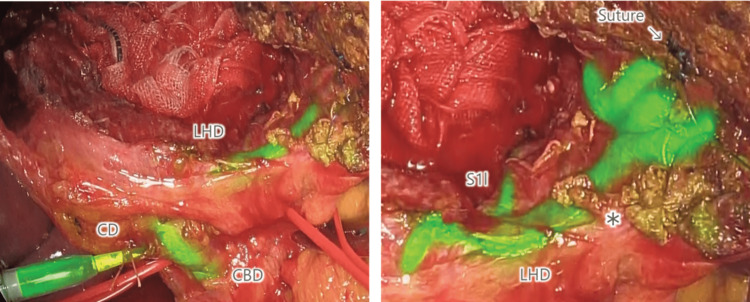
Intraoperative cholangiography during open hepatectomy (segment 4) After enucleation of the large hemangioma in S4, ICG solution (0.025 mg/mL) is injected into the cystic duct. Fluorescence in the bile ducts is observed with NIR laparoscopy (1688AIM 4K platform (Stryker Corporation, Kalamazoo, Michigan, USA)). The extrahepatic bile ducts, the left hepatic duct, and the caudate lobe brunch (S1l) are clearly visualized. A part of the left hepatic duct on which a piece of hepatic tissue (asterisk) does not show fluorescence. CD: cystic duct; CBD: common bile duct; LHD: left hepatic duct; S1l: bile duct of the segment 1l; ICG: indocyanine green; NIR: near-infrared

A 64-year-old man with a large hemangioma (90 mm) close to the hilum underwent open surgery. After the enucleation of the tumor in segment four (S4), a broken bile duct end on the cut surface was sutured. ICG solution (0.025 mg/mL) was injected into the biliary tract for the leak test. The left hepatic duct and its branches were visualized (Figure 4), and no bile leakage was detected. Even a small piece of hepatic tissue on the biliary duct blocked fluorescence.

## Discussion

This study aimed to demonstrate the possibility of intraoperative FC by ICG intrabiliary injection with NIR laparoscopy during major hepatectomy. First, we determined the optimal conditions for ICG-FC as a preliminary ex vivo experiment. Subsequently, we performed intraoperative FC by intra-cystic-duct ICG injection with a laparoscopic NIR system as a clinical trial. We used laparoscopy systems from three different companies and obtained clear visualization of the intrahepatic bile ducts in the Glissonian sheath with every system. Furthermore, these NIR laparoscopies were effective in testing for bile leaks. This is the first report of real-time intraoperative FC in major hepatectomy with ICG-NIR laparoscopy. We anticipate that this method will help to avoid unexpected biliary injuries and improve safety in laparoscopic hepatobiliary surgery.

ICG fluorescence has been used in hepatobiliary surgery to visualize hepatic tumors, the border of the liver segments, and the biliary tract [3]. There are two ICG administration routes: intravenous and intrabiliary, and the intrabiliary can be divided into the intra-gall bladder, cystic duct, and bile drainage tube. For hepatic tumor imaging, ICG solution was injected intravenously several days before surgery. Injected ICG remained in and around the tumor, and the staining patterns differed according to the tumor characteristics (primary or metastatic) and the degree of differentiation [3]. For hepatic segment visualization, ICG was injected intravenously after clamping the Glissonian pedicle. The whole liver, except for the ischemic segment, showed ICG fluorescence [4].

There have been several reports of FC in cholecystectomy and hepatectomy (Table 1).

**Table 1:** Relevant reports regarding ICG fluorescent cholangiography during hepatobiliary surgery EHBD: extrahepatic bile duct; IHBD: intrahepatic bile duct; LC: laparoscopic cholecystectomy; lap.: laparoscopic; Hx: hepatectomy; IB: intrabiliary injection; IV: intravenous injection; CD: cystic duct; GB: gall bladder; DT: drainage tube * Accessory bile duct

Author	Year	Operation	Route	ICG dose	Device	Detected		Other
						EHBD	IHBD	
Ishizawa et al. [5]	2009	Open Hx	IB-CD	0.025 mg/ml	PDE (Hamamatsu P)	✔	✔	-
Aoki et al. [6]	2010	LC	IV	12.5 mg	Prototype	✔	-	-
Ishizawa et al. [7]	2010	LC	IV	2.5 mg	Prototype	✔	-	accessory *
Sakaguchi et al. [8]	2010	Open Hx	IB-CD	0.05 mg/ml	PDE (Hamamatsu P)	-	-	leak test
Mizuno et al. [9]	2010	Open Hx	IB-CD	0.025 mg/ml	PDE (Hamamatsu P)	✔	-	accessory *
Kaibori et al. [10]	2011	Open Hx	IB-CD	2.5 mg/ml	PDE (Hamamatsu P)	-	-	leak test
Kawaguchi et al. [11]	2011	Open Hx	IB-CD	0.025 mg/ml	HyperEye Medical System (Mizuho Medical Co. Ltd., Tokyo, Japan)	✔	-	-
Kawaguchi et al. [12]	2015	Lap Hx	IV	0.025 mg	Olympus Medical Systems	✔	-	-
Graves et al. [13]	2017	LC	IB-GB	0.25 mg	Stryker Corporation	✔	-	-
Shibata et al. [14]	2021	Open Hx	IB-GB or DT	0.025 mg/ml	Not described	✔	-	-
Case 1	2021	Lap Hx	IB-CD	0.01 mg/ml	VISERA ELITE II (Olympus Medical Systems)	✔	✔	leak test
Case 2	2021	Lap Hx	IB-CD	0.025 mg/ml	IMAGE1 S™ 4U RUBINA (KARL STORZ)	✔	✔	leak test
Case 3	2021	Lap Hx	IB-CD	0.05 mg/ml	1688AIM 4 K platform (Stryker Corporation)	✔	✔	leak test

Most of these FCs were performed with ICG-IV, although there are few reports examining the intrabiliary method in hepatectomy. In the ex vivo experiments, we measured the fluorescence intensity of the diluted ICG solution. The fluorescence was revealed to be low if the ICG concentration was too high (≥ 0.25 mg/mL) or too low (≤ 0.005 mg/mL). When mixed with an equal amount of bile, ICG solutions with more than 0.05 mg/mL concentration showed strong fluorescence twice as high as the control (0.01 mg/mL). The fluorescence sensitivity varied according to the distance between the camera and the object, the angle of view, and the location of the object in the field of view (Video 1). According to the results of the ex vivo experiment, the reasons why the intrabiliary FC was not clinically used were assumed to be that the fluorescent intensity was low because the injected ICG concentration was too high, ICG and bile were not mixed sufficiently, and the NIR camera was not set in front of the object or far from it. However, when leaked ICG solution was mixed with bile, blood, or lymphatic fluid on the hepatic cut surface, strong fluorescence was observed, even under insufficient observation conditions. Therefore, ICG leak tests have been reported after hepatectomy. At one point, a high concentration of ICG spilled out onto the liver surface, and its glow persisted for an extended period during the procedure and could not be removed by several saline washes.

Ishizawa et al. successfully performed intrahepatic FC by intrabiliary ICG injection via the cystic duct (0.025 mg/mL) in open hepatectomy [5]. This method seemed helpful but was not generally used, likely because the infrared camera was too large and heavy to maintain a frontal view of the deep surgical field. Kawaguchi et al. reported intraoperative FC by intravenous ICG injection (0.025 mg after intubation) in laparoscopic hepatectomy [12]. They successfully visualized extrahepatic bile ducts in the hepatoduodenal ligament. As we demonstrated, direct intrabiliary ICG injection and the development of laparoscopic devices enable intrahepatic FC. Intrabiliary FC can be concomitantly performed with both intravenous tumor imaging and intravenous hepatic segment imaging if ICG is administered with proper timing.

We successfully visualized the bile ducts in the Glissonian sheath using three different laparoscopic systems. However, even a small piece of hepatic tissue overlying the bile duct was shown to have the ability to prevent visualization. The major limitation of FC is the low tissue penetration depth of NIR light. Although Kim et al. reported that they identified lymph nodes 1 cm below the skin surface of pigs using a NIR camera [15], our experiments were only able to visualize the bile duct in the exposed Glissonian sheath and under the thin tissue of the hepatoduodenal ligament. It is assumed that the excitation light of laparoscopy is less powerful than that of the open surgery system. Therefore, it is necessary for clear NIR-FC to remove the hepatic tissue surrounding the Glissonian sheath.

In this study, we tested several concentrations of ICG solution and three different laparoscopic systems. In case one, intrahepatic bile ducts were detected with ICG solution at a concentration of 0.01 mg/mL. Furthermore, a more precise observation was possible when the laparoscope moved closely at a right angle to the object. In another case, laparoscopic cholangiography with 0.025 mg/mL ICG solution seemed to produce a higher contrast image than 0.01 mg/mL solution (data not shown). It is difficult to compare different ICG concentrations in one case because the ICG glow remains for a long time after intrabiliary injection. In some cases of bile leakage, the ICG solution at a higher concentration (≥ 0.025 mg/mL) was difficult to wash when it adhered to the surrounding tissue. In this study, we did not determine the universal ICG concentration for both FC and leak tests in the three different laparoscopic systems. To date, ICG solution should be evaluated from a low concentration (0.01 mg/mL) to a higher concentration (up to 0.05 mg/mL) according to the purpose of the fluorography. Quantification of image contrast and optimal conditions of the ICG solution should be examined individually according to the purpose and devices.

Intrabiliary injection requires tube placement into the cystic duct. It is sometimes difficult and requires proficiency in intubation and fixation using laparoscopic devices. Furthermore, the tube easily comes off when strong infusion pressure is applied. We often use a 5 Fr (1.7 mm in outside diameter) feeding tube, which is fixed by a double ligature with an elastic suture. While the intrabiliary FC enables real-time biliary visualization during a surgical operation, it requires time-consuming preparation. Thus, it should be applied when the benefit is higher than the preparation cost. For example, intrabiliary FC is assumed to be effective for laparoscopic anatomical hepatectomy, especially anterior segmentectomy with a Glissonian pedicle transection, because it is at risk for biliary injury of the posterior segment branches, and real-time visualization of these biliary tracts may prevent unexpected injury.

## Conclusions

Our experiments proved that it is possible to perform real-time intraoperative FC using ICG-intrabiliary injection and NIR laparoscopy. Our ultimate purpose is to improve the safety of laparoscopic major hepatectomy. Further well-designed clinical trials are needed to validate our findings. Furthermore, the intrabiliary administration procedure must be simplified to make ICG-FC widely available in hepatobiliary surgery.

## Figures and Tables

**Figure 5:**
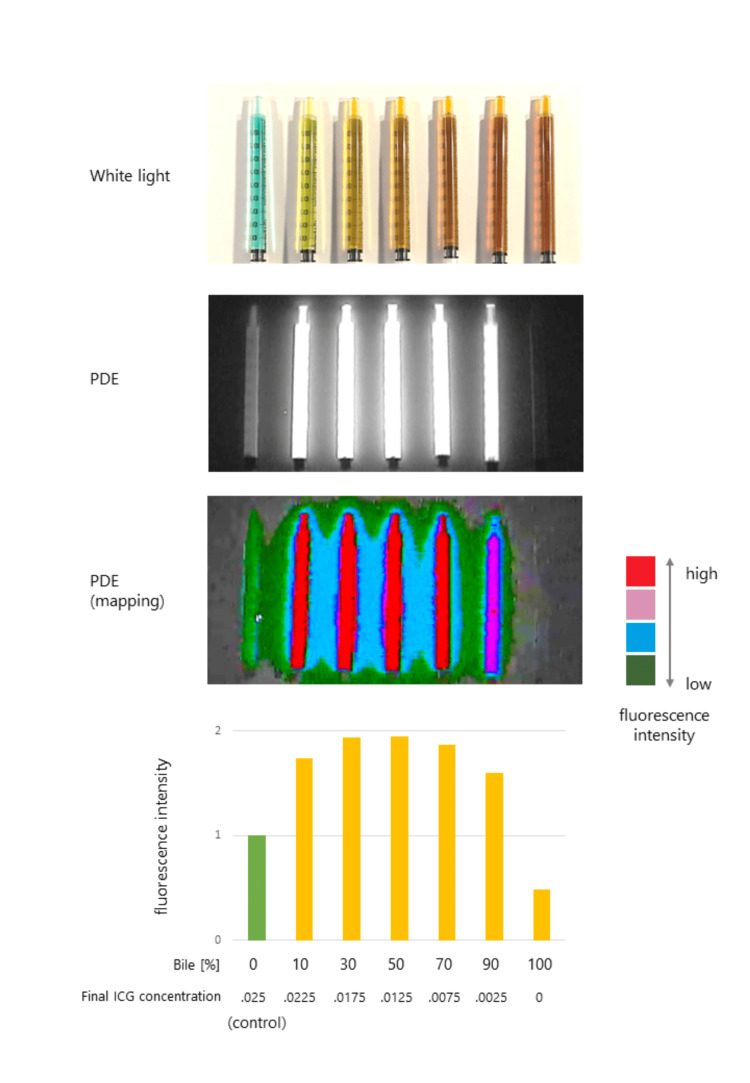
ICG solution and bile were mixed in varying proportions ICG solution (0.025 mg/mL) and bile were mixed in varying proportions. The fluorescence of the 0.025 mg/mL ICG sample was set as a standard. The fluorescence intensity of each sample was quantified relative to the standard value. PDE: photodynamic eye-neo

## Appendices

Appendix one 
